# Magnesium sulphate and amiodarone prophylaxis for prevention of postoperative arrhythmia in coronary by-pass operations

**DOI:** 10.1186/1749-8090-4-8

**Published:** 2009-02-20

**Authors:** Osman Tiryakioglu, Sinan Demirtas, Hasan Ari, Selma Kenar Tiryakioglu, Kagan Huysal, Ozer Selimoglu, Ahmet Ozyazicioglu

**Affiliations:** 1 Bursa Yuksek İhtisas Education and Research Hospital, Department of Cardiovascular Surgery, Bursa, Turkey; 2 Bursa Yuksek İhtisas Education and Research Hospital, Department of Cardiology, Bursa, Turkey; 3 Bursa Yuksek İhtisas Education and Research Hospital, Department of Biochemistry, Bursa, Turkey; 4 Medical Park Hospital, İstanbul, Turkey

## Abstract

**Background:**

The aim of this study was to investigate the use of prophylactic magnesium sulphate and amiodarone in treating arrhythmias that may occur following coronary bypass grafting operations.

**Methods:**

The study population consisted of 192 consecutive patients who were undergoing coronary artery bypass grafting (CABG). Sixty-four patients were given 3 g of magnesium sulphate (MgSO4) [20 ml = 24.32 mEq/L Mg^+2^] in 100 cc of isotonic 0.9% solution over 2 hours intravenously at the following times: 12 hours prior to the operation, immediately following the operation, and on postoperative days 1, 2, and 3 (Group 1). Another group of 64 patients was given a preoperative infusion of amiodarone (1200 mg) on first post-operative day (Group 2). After the operation amiodarone was administered orally at a dose of 600 mg/day. Sixty-four patients in group 3 (control group) had 100 cc. isotonic 0.9% as placebo, during the same time periods.

**Results:**

In the postoperative period, the magnesium values were significantly higher in Group 1 than in Group 2 for all measurements. The use of amiodarone for total arrhythmia was significantly more effective than prophylactic treatment with magnesium sulphate (p = 0.015). There was no difference between the two drugs in preventing supraventricular arrhythmia, although amiodarone significantly delayed the revealing time of atrial fibrillation (p = 0.026). Ventricular arrhythmia, in the form of ventricular extra systole, was more common in the magnesium prophylaxis group. The two groups showed no significant differences in other operative or postoperative measurements. No side effects of the drugs were observed.

**Conclusion:**

Prophylactic use of magnesium sulphate and amiodarone are both effective at preventing arrhythmia that may occur following coronary by-pass operations. Magnesium sulphate should be used in prophylactic treatment since it may decrease arrhythmia at low doses. If arrhythmia should occur despite this treatment, intervention with amiodarone may be preferable.

## Background

Postoperative rhythm disorders are a serious complication of open-heart surgery. The incidence of postoperative supraventricular arrhythmias has been reported to be 11–54%, and the incidence of ventricular arrhythmia to be 1.8–13%. [1]. Arrhythmias are frequently seen during the first 48 hours following open-heart surgery, necessitating effective postoperative monitoring. Knowledge of what causes the development and progression of arrhythmia in a postoperative patient may allow us to reduce the use of pharmacological and electrical procedures aimed at reducing the ventricular rate or ensuring normal sinus rhythm.

The large majority of arrhythmia cases in coronary artery bypass surgery (CABG), except insufficient revascularization, can be controlled by electrical cardio versions. However, most medications currently used include beta-blockers or negative inotropics and some of them increase myocardial oxygen consumption as a side effect. The decrease in the concentrations of ions such as potassium and magnesium during the postoperative period is an important factor in most of the cases of arrhythmia that do not have organic causes (e.g. insufficient revascularization and graft thrombosis). Identifying and detecting these ionic imbalances may protect the heart from the side effects of anti-arrhythmia treatments. Among the physiological ions, magnesium plays an important role in preserving cardiac rhythm by stabilizing membrane function [1-3].

The aim of our study was to compare the effects of magnesium sulphate and amiodarone in the prevention and treatment of ventricular and supraventricular arrhythmia following CABG.

## Methods

The study was performed on 192 patients who had undergone successful CABG. The local Ethics Committee approved of the study, and personal informed consent was taken.

Sixty-four patients were given 3 g of magnesium sulphate (MgSO4) [20 ml = 24.32 mEq/L Mg^+2^] in 100 cc of isotonic 0.9% solution intravenously for 2 hours at the following times: 12 hours prior to the operation, immediately following the operation, and on postoperative days 1, 2, and 3 (Group 1). The other group of 64 patients was given a preoperative amiodarone infusion of 1200 mg on the postoperative first day (Group 2). This dose was followed by an oral dose of 600 mg/day. Sixty-four patients in group 3 (control group) had 100 cc. isotonic 0.9% as placebo, during the same time periods.

During the same postoperative period, K^+ ^replacements [in the form of glucose-insulin-potassium (GIK) solution (500 cc of 5% dextrose + 90 meq K^+ ^+ 12 U crystallized insulin)] and Ca^+2 ^replacements (1 g Ca-gluconate in 100 cc of isotonic 0.9% solution) were administered in order to maintain a K^+ ^concentration of at least 3.5–5.5 meq/L, and a Ca^+2 ^concentration of at least 8–9 meq/L. In order to standardize the groups, patients were grouped based on the following parameters: age, gender, use of calcium channel blocker, use of beta-blocker, presence of diabetes, presence of renal disorder, presence of chronic obstructive pulmonary disease (COPD), history of non-cardiac operations, history of myocardial infarction (MI), classification of New York Heart Association (NYHA), end diastolic pressure (EDP), cross-clamp time, cardiopulmonary bypass durations, and coronary bypass count (Table 1).

**Table 1:** Preoperative and demographic characteristics of the patients in the study sample.

	Group 1 (Magnesium, n = 64)	Group 2 (Amiodarone, n = 64)	Group 3 (Control, n = 64)	P value
Age (years)	58 ± 8	61 ± 6	57.6 ± 8.8	NS

Sex (M/F)	40/24	42/22	52/8	NS

History of MI	32 (50%)	30 (47%)	30 (47%)	NS

Hypertension	23 (36%)	21 (33%)	23 (36%)	NS

Smoking	23 (36%)	16 (25%)	23 (36%)	NS

COPD	7 (11%)	8 (12. 5%)	8 (12. 5%)	NS

PTCA-Stent	4 (6.2%)	6 (9.4%)	4 (6.2%)	NS

Preoperative use of beta blocker	21 (33%)	23 (36%)	21 (33%)	NS

BSA	1.79 ± 0.2	1.78 ± 0.3	1.78 ± 0.3	NS

LVEDP(mmHg)	13.6 ± 4.2	16.9 ± 6.5	14.6 ± 4.4	NS

Left ventricle EF (mean %)	45 ± 4	43 ± 3	44 ± 4	NS

Diabetes Mellitus	20 (31%)	18 (28%)	18 (28%)	NS

MI, myocardial infarction; COPD, chronical obstructive pulmonary disease; NYHA, New York Heart Association; PTCA, percutan transluminal coronary angioplasty; BSA, body surface area; LVEDP, left ventricle end diastolic pressure; NS, not significant (p > 0.05).

Anti-arrhythmia drugs used in the preoperative period were halted 24 hours before the operation. Twelve derivation electrocardiographies of the patients were taken on the morning of the operation day and patients showing arrhythmia or ischemia were excluded from the study.

## Operational procedure

Midazolam (0.03 mg/kg), hypnomidate (0.3 mg/kg), phentanyl (5 μg/kg), and pancuronium bromide (0.1 mg/kg) were used for anesthesia. When continuation was needed, 5 μg/kg of phentanyl and 0.05 mg/kg of pancuronium or vecuronium bromide were used.

All patients were subjected to standard median sternotomy. The left internal mammarian artery (LIMA) and vena saphena magna from the lower extremity were dissected and prepared. The arteria radialis was used for two cases.

The LIMA graft was anastomosed to the left anterior descending coronary artery (LAD) in all cases. With the arterial cannulation from the aorta ascending and two-stage venous cannulation from right atrial auricular, the cardio-pulmonary bypass was introduced. A cardioplegia cannula was placed in the aortic root and all the patients were given cardioplegia by the anterograde way. A supply of oxygenated blood was prepared throughout the operation, and the cardioplegic fluid prepared in this way was administered using a "warm induction" procedure in order to cause diastolic arrest. A total volume of 10 ml/kg of cardioplegic fluid at a temperature of 37°C was introduced into the heart through the aortic root. Cardioplegia was periodically continued for periods of 15 minutes after cooling. Warming was initiated as the LIMA-LAD anastomosis was performed. Before the cross-clamp was removed, a final dose of "hot shot" cardioplegic fluid (10 ml/kg, 37°C) was delivered in order to control reperfusion and prevent any reperfusion damage. Moderate hypothermia (28°C) was maintained throughout the operation.

None of the patients in any group received any anti-arrhythmia protocol other than those described.

Magnesium blood ion analysis: plasma Mg^+2 ^levels were determined by spectrophotometric analysis of venous blood samples taken immediately after the operation and on postoperative days 1, 2, and 3. Total Mg^+2 ^levels were recorded. At the same time, levels of Na^+^, K^+^, and Ca^+2 ^were also determined, and their insufficiencies replaced in the patients.

Liver function tests (SGOT, SGPT), urea and creatinine, creatine kinase (CK), and creatine kinase MB (CK-MB) were measured preoperatively, at preoperative time 0, and postoperatively on days 1, 2, and 3. Cardiac troponin (cTn-T) was measured preoperatively, and at 12 and 24 hours postoperatively in all cases.

Follow-up of cardiac rhythm and arrhythmia: Patients were monitored during the preoperative period and for at least 24 hours postoperatively. The rhythm follow-up of cases was confirmed by ECG at 0, 6, and 12 hours postoperatively, as well as on postoperative days 1, 2, and 3.

Cases of ventricular tachycardia were medicated if they caused multifocal or hypertensive at a frequency of more than 10 per minute. Bigeminal and trigeminal rhythms and ventricular early beats that persisted long less 30 seconds and that did not include the properties mentioned above were not medicated. In the case of supraventricular arrhythmia, if the ion balance and K^+ ^levels became normal but there was fast ventricular response AF, speed-limiting anti-arrhythmic agents were used.

Exclusion criteria for this study were the following: Preoperative renal insufficiency, requirement of a second open-heart operation, left ventricular aneurism, additional cardiac pathology, and ischemia at the beginning of the operation (angina and ST alterations), off-pump CABS, and single-vessel disorders.

## Results and discussion

The groups were created based on several variables such that they would not have significant differences in their demographic or preoperative profiles. The groups did not show a statistical difference in their mean left ventricle ejection fraction (EF) (p > 0.05) (Table 1).

LIMA was used for LDA bypass in all patients. The radial artery was used for two patients, while all other anastomoses were performed using vena saphena grafts. The mean number of grafts used was 3.1 ± 1.2 in Group 1, 3.2 ± 1.4 in Group 2 and 3.1 ± 1.4 in Group 3, and these means were not significantly different. No significant difference was observed between groups based on mean cross clamp duration, perfusion duration, or use of inotropics. Similarly, mean transfusion amount, duration of intubation, duration of stay in the intensive care unit, and overall length of hospital stay did not differ significantly between the groups (Table 2).

**Table 2:** Preoperative and postoperative patient data.

	Group 1	Group 2	Group 3	p
LIMA usage	64 (100%)	64 (100%)	64 (100%)	NS

Saphenous graft usage	134	138	140	NS

Radial artery usage	1 (1.5%)	1 (1.5%)	-	NS

Mean number of grafts	3.1 ± 1.2	3.2 ± 1.4	3.1 ± 1.4	NS

Blood transfusion (units)	2 ± 1.5	2 ± 1.8	2 ± 1.8	NS

Duration of intubation (hours)	16.6 ± 3.3	15.2 ± 3.2	14.4 ± 3.5	NS

Duration of stay in intensive care unit	50.3 ± 5.3	49.2 ± 2.4	50 ± 5.5	NS

Discharge time (days)	7 ± 1	8 ± 2	7 ± 1	NS

Inotrope support	24 (37.5%)	20 (31.2%)	24 (37.5%)	NS

Cross aortic clamp time (min)	82 ± 26	85 ± 22	80 ± 25	NS

Perfusion time (min)	104 ± 44	106 ± 30	100 ± 41	NS

NS, not significant (p > 0.05).

Pre- and postoperative magnesium levels did not differ between the three groups. In Group 1, all the measured values showed a significant increase due to magnesium sulphate prophylaxis treatment during the postoperative period (day 0, p = 0.001; day 1, p = 0.02; day 2, p = 0.02; day 3, p < 0.05 in other groups). However, a decrease was observed in the level of magnesium following CABG during the preoperative period. Potassium and calcium levels were similar in both groups for in all measurements taken to assess whether replacement therapy was needed (Table 3).

**Table 3:** Magnesium values of patients

		Group 1	Group 2	Group 3	P value
Magnesium (mg/dL)	Preoperative	1.9 ± 0.3	1.9 ± 0.2	2.1 ± 0.3	NS

	Perioperative	1.7 ± 0.2	1.7 ± 0.3	1.8 ± 0.3	NS
	
	0*	3.6 ± 0.4	1.5 ± 0.2	1.8 ± 0.3	0.001**
	
	1*	3.1 ± 0.7	1.6 ± 0.2	1.7 ± 0.3	0.02**
	
	2*	3.0 ± 0.6	1.6 ± 0.3	1.7 ± 0.3	0.02**
	
	3*	2.9 ± 0.6	1.7 ± 0.2	1.8 ± 0.3	0.04**

*Postoperative days; ** Significant (Difference between Group 1 and other groups). NS, not significant (p > 0.05).

Values of CK-MB and cardiac troponin t (cTnT) did not increase significantly for either group during the postoperative period (Table 4).

**Table 4:** Enzyme values of patients.

		Group 1	Group 2	Group 3	P value
CK-MB (U/L)	Preoperative	29 ± 2.5	17 ± 2.1	17 ± 2.5	NS

	12 hr	53 ± 4.1	108 ± 5.5	83 ± 3.7	NS

	24 hr	74 ± 3.6	77 ± 3.7	63 ± 4.1	NS

	3 day	42 ± 2.3	47 ± 3.2	44 ± 2.3	NS

cTn-T (ng/mL)	Preoperative	1.23 ± 0.2	1.91 ± 0.3	1,61 ± 0.4	NS

	12 hr	2.11 ± 0.3	2.45 ± 0.4	2,53 ± 0.3	NS

	24 hr	1.31 ± 0.4	1.54 ± 0.2	1,27 ± 0.4	NS

	3 day	0.9 ± 0.2	0.7 ± 0.1	0.8 ± 0.2	NS

CK-MB, creatine kinase-MB; cTn-T, cardiac troponin-T; NS, Not Significant (p > 0.05).

When postoperative arrhythmias were investigated, the total arrhythmia count of all patients was 25 (19.5%). Of these, 17 (13.35%) were supraventricular, and eight (6.25%) were ventricular. All the cases of supraventricular arrhythmia involved atrial fibrillation, and two patients in the magnesium group showed pre-POAF atrial extrasystoles. No ventricular arrhythmia was detected in the amiodarone group. Arrhythmias in the form of extrasystoles were seen in eight patients of the magnesium group. When the arrhythmias were more than 10 per minute, lidocaine and metoprolol were administered. All eight cases were detected at the end of the first dose of anti-arrhythmic treatment. These cases were given additional postoperative treatment of metoprolol (50 mg/day).

The AF that occurred postoperatively in the magnesium group was treated with amiodarone infusion (900 mg/day loading, 600 mg/day oral continuation), and all patients recovered normal sinus rhythm. In the amiodarone prophylaxis group, patients were given magnesium (3 g/day), although two of seven patients (28.5%) recovered normal sinus rhythm, while the other five (71.5%) remained at AF. These latter patients continued to receive amiodarone treatment, and no additional anti-arrhythmic medication was given. On average, patients recovered normal sinus rhythm by postoperative day 6 (Table 5).

**Table 5:** Postoperative arrhythmia observed in the patient sample.

		Group 1 (n = 64)	Group 2 (n = 64)	Group 3 (n = 64)	P value
Postoperative arrhythmia		18 (28.1%)	7 (10.9%)	30 (46%)	0.015*

Supraventricular arrhythmia		10 (16.7%)	7 (10.9%)	20 (31%)	NS

	Atrial extrasystole	2 (3.3%)	0	0	NS

	Atrial fibrillation	10 (16.7%)	7 (10.9%)	20 (37.3%)	NS

AF exit time (hours)		49 ± 15	123 ± 27	48 ± 17	0.026*

Ventricular arrhythmia		8 (12.5%)	0	10 (%15)	0.001*

	Ventricular extrasystole	8 (12.5%)	0	10 (%15)	0.001*

* Significant (Difference between Group 1 and Group 2).

## Discussion

AF is an undesired but frequent complication of CABG observed in 10–40% of cases. It prolongs a patient's stay in the intensive care unit or hospital, and it disturbs a patient's comfort. In addition, AF postpones full recovery after CABG. Thus, many drugs have been used prophylactically in order to prevent POAF: beta-blockers, calcium channel blockers, magnesium sulphate, and amiadarone. All of these drugs have different indications or counter indications [2-4].

Reducing the frequency of arrhythmia of a patient after CABG reduces both the duration of hospitalization and medical costs. Studies have shown that 25–30% of the patients had temporary supraventricular arrhythmia attacks despite using β-blockers. As is widely known, β-blockers are not indicated for those with poor ventricle function who frequently demonstrate arrhythmia. Instead, amiodarone is recommended for these patients [4].

However, the sufficiency and reliability of amiodarone in preventing arrhythmia is controversial. Oral amiodarone is known to be insufficient for preventing postoperative arrhythmia, but sometimes the preoperative loading dose can prove sufficient. Amiodarone given during the preoperative period has been reported to react with anesthetic agents and cause pulmonary dysfunction, hypotension, hepatic dysfunction, and low heart flow. However, Daoud et al. have reported that preoperative amiodarone use does not increase the risk of postoperative mortality and morbidity [4,5]. Similarly, we did not observe any side effects in the amiodarone group in the present study. According to our observations, both drugs are safe.

Our results indicate that amiodarone is significantly more effective than magnesium sulphate in treating total arrhythmia. However, this may be because the ventricular extrasystoles frequently disappear in the absence of any medication. The two groups showed no differences in the rate of recovery from supraventricular arrhythmia.

Magnesium is a cation that functions by lengthening the refractory period at the atrioventricular node. Thus, magnesium likely has an important role in preventing and treating atrial fibrillation, especially considering that serum magnesium levels below 0.8 mmol/L trigger atrial fibrillation. Thus, dose loading with magnesium can prevent the arrhythmia caused by the postoperative decrease in this caution.

In our study we observed that the serum magnesium levels were low in the postoperative period, although never below 0.8 mmol/L [2].

Many comparative studies and meta-analyses have been published on this issue. In a meta-analysis evaluating atrial fibrillation after CABG, the frequency of POAF was determined to be 32.3%. POAF is frequently concomitant with renal insufficiency and infection. In this meta-analysis, preoperative COPD and older age were determined to be risk factors for POAF. The investigators concluded that atrial fibrillation is an important complication of CABG, and they suggested preventing it by administering β-blockers and ACE inhibitors [6].

Çağlı et al. performed a study that combined amiodarone and MgSO_4_, and they concluded that together these agents are tolerable and work more effectively in high-risk patients than they do on their own [7]. A different study showed that older age and lower magnesium plasma levels are the most important risk factors of POAF. This same study reported that amiodarone was effective for POAF, whereas magnesium prophylaxis had no effect [5-8]. In our study, we administered amiodarone to patients with AF despite magnesium prophylaxis; we administered amiodarone orally, following an initial loading dose.

Studies have examined the effects of medications other than prophylaxis following AF. Parenteral magnesium was reported to be superior to amiodarone in studies of acute atrial tachyarrhythmia [9]. Davey et al. have shown that magnesium sulphate slows the heart rate and prevents supraventricular arrhythmia. They also found that AF frequently returns to normal sinus rhythm in patients treated with magnesium sulphate. Lastly, they reported that magnesium-related hypotension and bradycardia are potential risk factors for AF [10].

In a meta-analysis evaluating eight different clinical studies, the use of different doses of magnesium sulphate, placebo, amiodarone, and diltiazem following atrial fibrillation was examined. During the first hour following its application, magnesium was found to be superior to other anti-arrhythmics for controlling ventricular speed (patients with a heartbeat of less than 100/min). Magnesium was also significantly more effective than placebo or diltiazem for restoring normal sinus rhythm within the first 15 hours. As a result, magnesium sulphate effectively controls heart rhythm following AF [11]. In another study, magnesium was found to be effective at preventing postoperative ventricular arrhythmia in a dose-dependent manner [12].

## Conclusion

The types of patients examined in this study require treatment for their atrial fibrillation. Our study shows that both amiodarone and magnesium sulphate work safely and sufficiently rapidly for this purpose. Our clinic already uses amiodarone frequently, and this study shows that magnesium is also effective at treating postoperative arrhythmia. In fact, using magnesium may avoid low postoperative levels of magnesium and its concomitant side effects, as well as postoperative atrial fibrillation. Thus, we suggest routine magnesium sulphate administration in patients undergoing open-heart surgery.

## Competing interests

The authors declare that they have no competing interests.

## Authors' contributions

OT: Designed the study, coordinated it, and performed statistical analysis. SD: Conceived the study and participated in its design. HA: Designed the study and performed the echocardiographic study and performed statistical analysis. SKT: Designed the study and performed the echocardiographic study. KH: Performed biochemical analysis. OS: Performed statistical analysis. AO: Coordinated the study.
